# The First 500 Registrations to the Research Registry^®^: Advancing Registration of Under-Registered Study Types

**DOI:** 10.3389/fsurg.2016.00050

**Published:** 2016-09-16

**Authors:** Riaz Agha, Alexander J. Fowler, Christopher Limb, Yasser Al Omran, Harkiran Sagoo, Kiron Koshy, Daniyal J. Jafree, Mohammed Omer Anwar, Peter McCullogh, Dennis Paul Orgill

**Affiliations:** ^1^Balliol College, University of Oxford, Oxford, UK; ^2^Department of Plastic Surgery, Guy’s and St. Thomas’ NHS Foundation Trust, London, UK; ^3^Department of Medicine, Guy’s and St. Thomas’ NHS Foundation Trust, London, UK; ^4^St. Helier Hospital, Surrey, UK; ^5^Royal Berkshire Hospital, Reading, UK; ^6^GKT School of Medical Education, Kings College London, London, UK; ^7^UCL Medical School, University College London, London, UK; ^8^Queen Mary University of London, London, UK; ^9^Nuffield Department of Surgery, University of Oxford, Oxford, UK; ^10^Harvard Medical School, Boston, MA, USA

**Keywords:** Helsinki Declaration, evidence-based medicine, registration, research design, databases

## Abstract

**Introduction:**

The Declaration of Helsinki 2013 encourages the registration of all research studies involving human participants. However, emphasis has been placed on prospective clinical trials, and it is estimated that only 10% of observational studies are registered. In response, Research Registry^®^^1^ was launched in February 2015; a retrospectively curated registry that is free and easy to use. Research Registry^®^ enables prospective or retrospective registration of studies, including those study types that cannot be registered on existing registries. In this study, we describe the first 500 registrations on Research Registry^®^.

**Methods:**

Since the launch of Research Registry^®^ in February 2015, data of registrations have been collected, including type of studies registered, country of origin, and data curation activity. Inappropriate registrations, such as duplicates, were identified by the data curation process. These were removed from the database or modified as required. A quality score was assigned for each registration, based on Sir Austin Bradford Hill’s criteria on what research studies should convey. Changes in quality scores over time were assessed.

**Results:**

A total of 500 studies were registered on Research Registry^®^ from February 2015 to October 2015, with a total of 1.7 million patients enrolled. The most common study types were retrospective cohort studies (37.2%), case series (14.8%), and first-in-man case reports (10.4%). Registrations were received from 57 different countries; the most submissions were received from Turkey, followed by China and the United Kingdom. Retrospective data curation identified 80 studies that were initially registered as the incorrect study type, and were subsequently correct. The Kruskal–Wallis test identified a significant improvement in quality scores for registrations from February 2015 to October 2015 (*p* < 0.0001).

**Conclusions:**

Since its conception in February 2015, Research Registry^®^ has established itself as a new registry that is free, easy to use, and enables the registration of various study types, including observational studies and first-in-man case reports. Going forward, our plan is to continue developing Research Registry^®^ in line with user feedback and usability studies. We plan to further promote Research Registry^®^ to advance the cause of registration of research, to increase compliance with the Declaration of Helsinki 2013.

## Introduction

In 2008, the Declaration of Helsinki stated: “Every clinical trial must be registered in a publicly accessible database before recruitment of the first subject.” This core ethical guidance was updated in 2013 as follows: “Every research study involving human subjects must be registered in a publicly accessible database before recruitment of the first subject.”

Registration of research studies is of great importance for the progress of medical research. There are a number of benefits to registering a study (1). For example, the problem of publication bias, whereby studies are performed but not reported, can be more accurately identified and dissuaded. Chapman and colleagues found that one in five surgical randomized controlled trials (RCT) are discontinued early, and one in three remains unpublished 2 years after their conclusion (2). This information is only available due to RCT registration. Another benefit of registering a study protocol, prior to conducting research, is the possibility to pre-specify research questions and outcomes, allowing peer-reviewers and readers to compare these to the published results. This is critical, as demonstrated by Page and colleagues, who identified that a striking 38% of systematic reviews have discrepancy in outcome reporting between protocols and published material (3). Moreover, registration of studies reduces duplication of work, a key source of waste in biomedical research (4).

The registration of clinical trials has been the focus of significant attention. However, while clinical trial registration has improved with time, there has been little improvement in observational studies, despite these making up a growing portion of the research landscape (5). It is estimated that only 10% of observational studies are registered at present on existing registries, and we have argued the need for a new registry. Many registries do not allow retrospective registration, have cost and time implications, and there is often a significant bureaucratic process to register studies (6). As a result, the Research Registry^®^ (www.researchregistry.com) was launched in February 2015.

**Figure 1:**
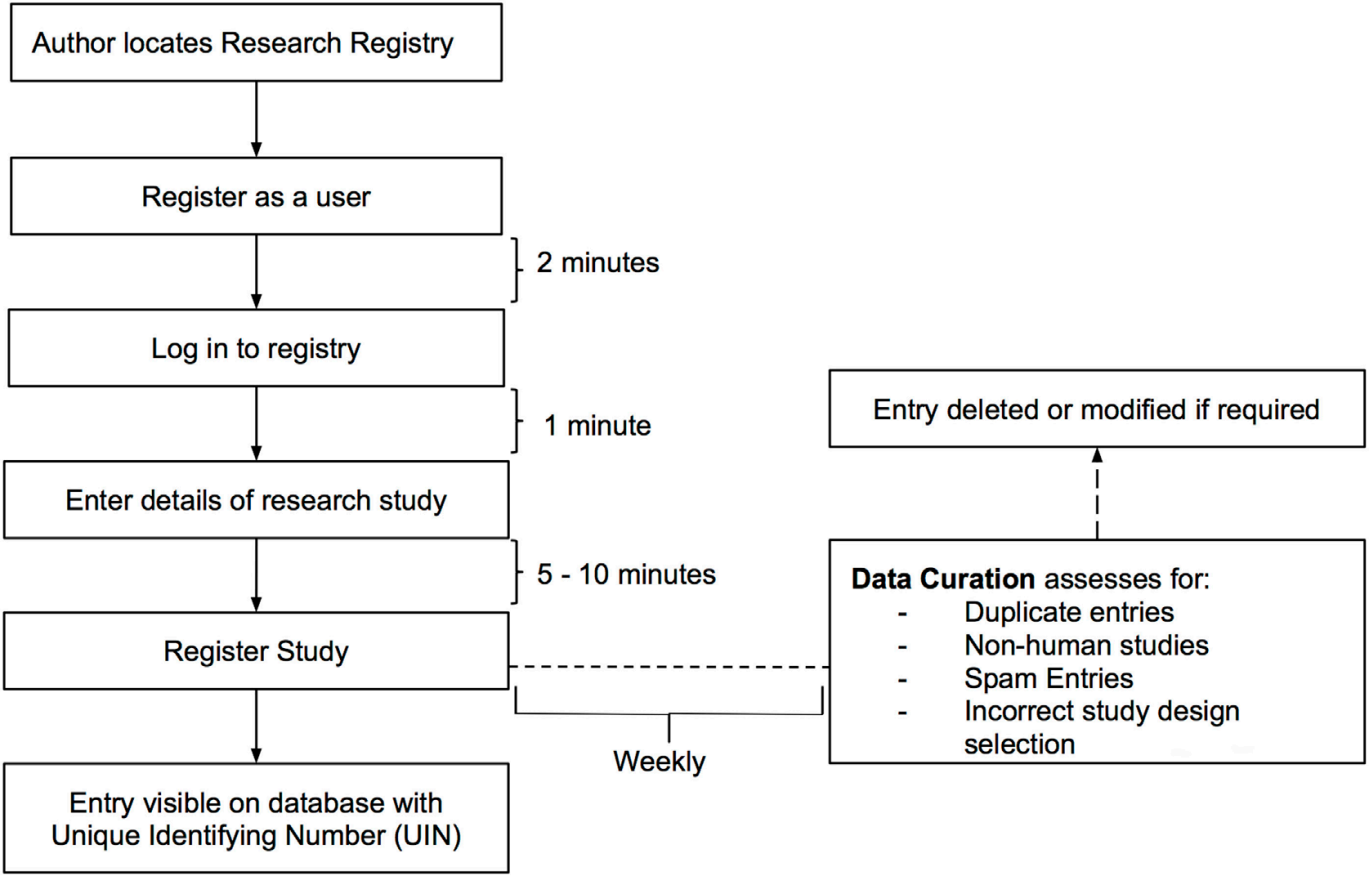
**Process of registration and data curation on the Research Registry^®^**.

**Figure 2:**
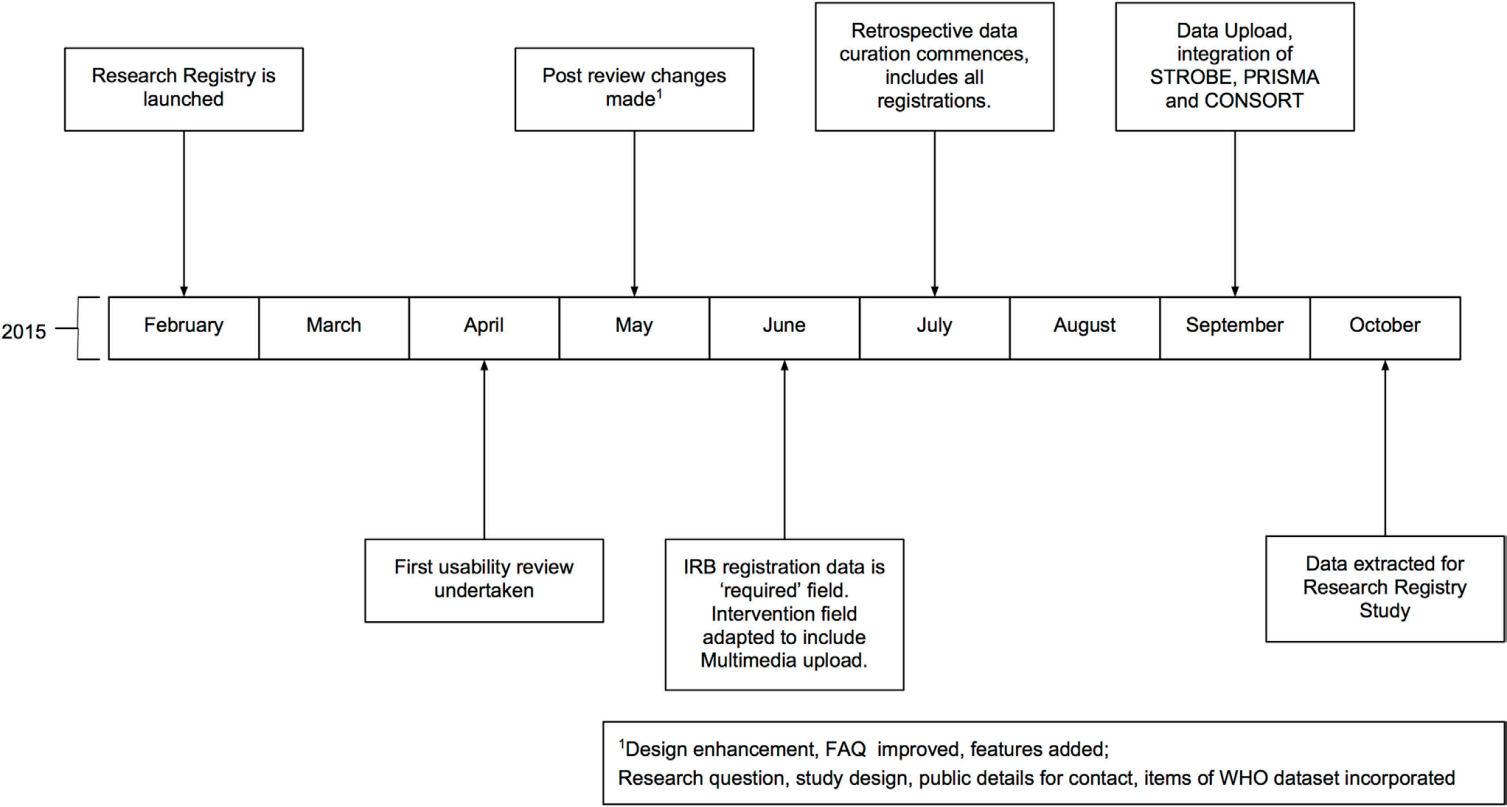
**Timeline of development of the Research Registry^®^**. All months are in 2015. IRB, Institutional Review Board; WHO, World Health Organization.

The Research Registry^®^ is funded through the International Journal of Surgery (IJS) Publishing Group, is free for all users to register and is open access. It enables free registration of any research study involving human participants, it takes a few minutes to register and allows prospective and retrospective registration. Researchers get immediate visibility of their registration, and a retrospective data curation process has been developed to remove or modify inappropriate registration (Figure 1). Data collected is in line with the World Health Organization (WHO) dataset for trial registration, and we have integrated elements of key reporting guidelines into the registration forms (7). Modifications to the registry have occurred following usability studies (Figure 2). This paper describes the first 500 registrations to the Research Registry^®^.

## Materials and Methods

### Descriptives of Registrations

This study was a retrospective database analysis of the Research Registry^®^, and involves no human participants, so was not registered. The Research Registry^®^ database (accessible at http://www.researchregistry.com/browse-the-registry.html) was extracted when 500 complete registrations were reached on 17th October 2015. The following characteristics were calculated based on the 500 extracted studies. The number of registrations per calendar month was calculated. The number of patients enrolled in registered studies was calculated with mean number of patients per registration. The study location, which is entered by authors upon registration, was also extracted. The types of studies registered and the number of registrations per study type were analyzed. Where authors unspecified study design by selecting “Other,” and then self-entered a study design, studies were allocated to the most appropriate study design after discussion between two curators (Alexander J. Fowler and Christopher Limb).

### Data Curation

Retrospective data curation is the means by which all registrations in the Research Registry^®^ are reviewed on a weekly basis. A standardized policy was followed by all curators on a rolling rota. Curators identified inappropriate registrations (Figure 1). Each curation team then generated a data curation report for that week. These reports included the number of registrations with identified problems, and the commonest reasons for inappropriate registration are provided. Data curation began prospectively in July 2015, but a full retrospective “sweep” was also performed to ensure the integrity of the database. Once inappropriate registrations had been highlighted, these were forwarded to and removed by the Director (Riaz Agha). At the end of the study period, after baseline data for the first 500 registrations to the Research Registry^®^ were obtained, data curation reports were retrieved and data extracted.

### Data Quality

The quality of data within the Research Registry^®^ was assessed using quality criteria that were developed alongside the registry. These are based on Sir Austin Bradford Hill’s criteria for what an account of a research study should convey (8), and each of the four key questions is linked directly to fields in the database (Table 1). Studies were scored out of nine using these quality criteria; receiving one point for each criterion met. Two teams of researchers (Christopher Limb/Mohammed Omer Anwar/Harkiran Sagoo and Yasser Al Omran/Kiron Koshy/Daniyal J. Jafree) scored each registration independently and compared results. Individual discrepancies in scoring were referred to the lead researcher for each group (Christopher Limb or Yasser Al Omran) for adjudication, and where consensus was not made, the decision was escalated to the lead author (Riaz Agha). The proportion of quality criteria met per registration was expressed as a percentage.

**Table 1:** **Quality indicator score for registrations**.

Question	Relevant area of registration form (http://www.researchregistry.com/browse-the-registry.html#home/addregistration/)
1. Who did the research?	Primary investigator
2. Where did they do the research?	Participating institutions Countries recruiting
3. Why did they do it?	Key questions and objectives
4. What did they do? This can be expanded to include the PICO items.	Patient population Intervention Control or comparator Primary outcomes (and secondary if used)
5. When did they do it?	Dates of enrollment

### Statistical Analysis

Descriptive data were calculated for the number of registrations, country of registration, and type of study. The number of patients was calculated by addition of the number of patients reported in the study registration for all 500 registrations. Inter-rater reliability was calculated for the quality criteria, and quality of registrations are presented on a month-by-month basis. An independent samples Kruskal–Wallis test was performed to ascertain if there was a significant difference between median month-by-month quality scores. All data were managed using Microsoft Excel 2013 (Microsoft, Redmond, VA, USA), statistical analysis was performed using IBM SPSS Statistics for Window, Version 22.0 (IBM Corp., Armond, NY, USA).

## Results

### Number of Study Registrations

**Figure 3:**
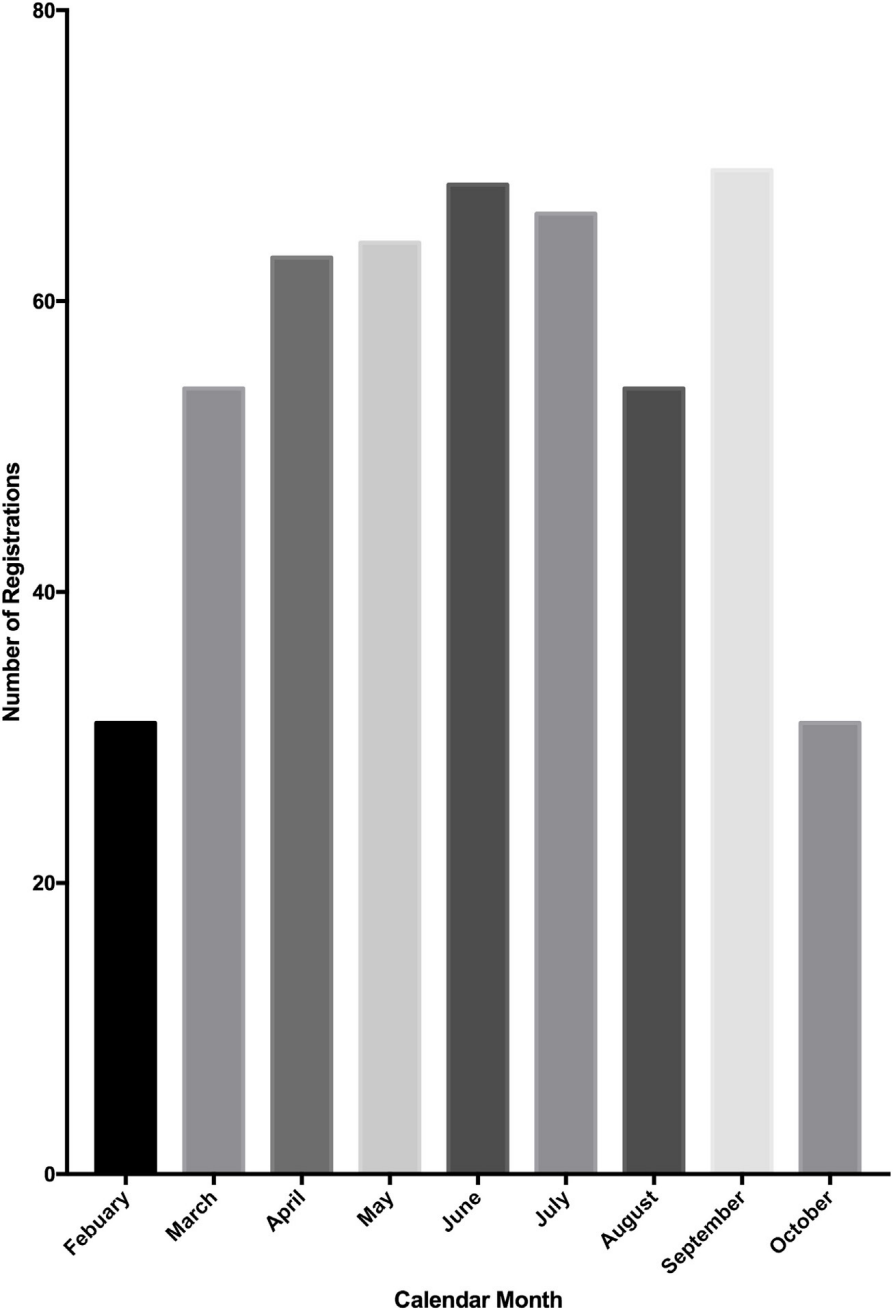
**Number of study registrations to the Research Registry^®^ per calendar month**. A total of 500 registrations were reached mid-way through October, so this graph represents only a portion of October’s registrations.

From the launch of the Research Registry^®^ in February 2015, a 9-month period passed until the registration of 500 research studies. In October 2015, the database was extracted for analysis. The number of registrations per calendar month was determined (Figure 3). There has been a mean growth of 6% in registrations per month since the launch of the registry in February 2015.

### Number of Included Patients

87.2% (436 of 500) registrations reported the number of patients included in their studies. Across these, a total of 1.77 million patients were enrolled. The median number of participants per study was 79 (inter quartile range: 30–200).

### Source of Registrations

**Figure 4:**
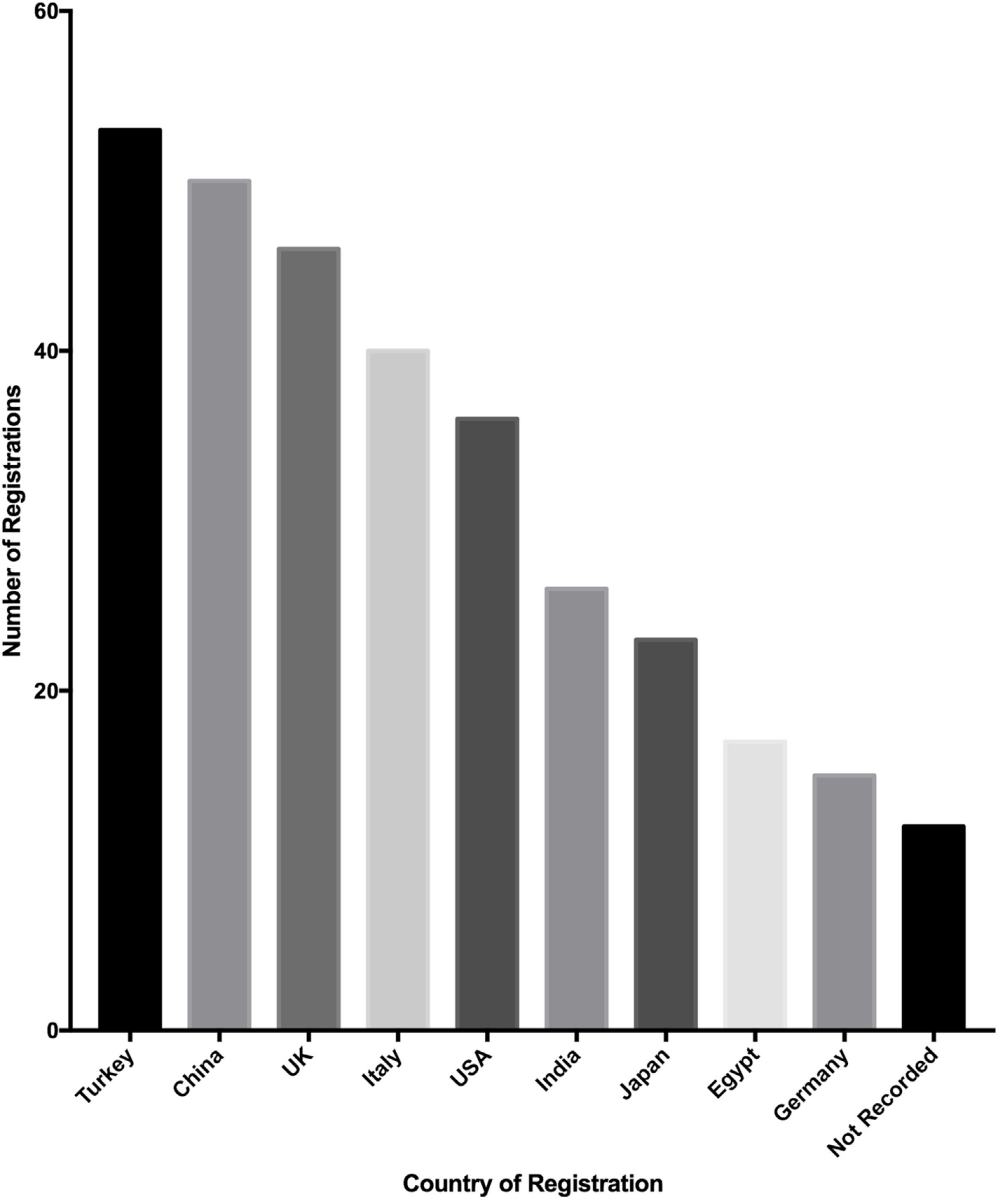
**Top 10 countries where studies were registered to the Research Registry^®^**.

Registrations originate from 57 countries, of which the top 10 countries from which registrations were analyzed (Figure 4). Turkey registered the most studies (52 of 500, 10.4%), followed by China (50 of 500, 10%) and the United Kingdom (46 of 500, 9.2%). The top 10 countries of registration made up 329 of 500 studies (65.8%) and the mean number of studies registered by these countries was 30 (SD = 14.9).

### Types of Studies Registered

**Figure 5:**
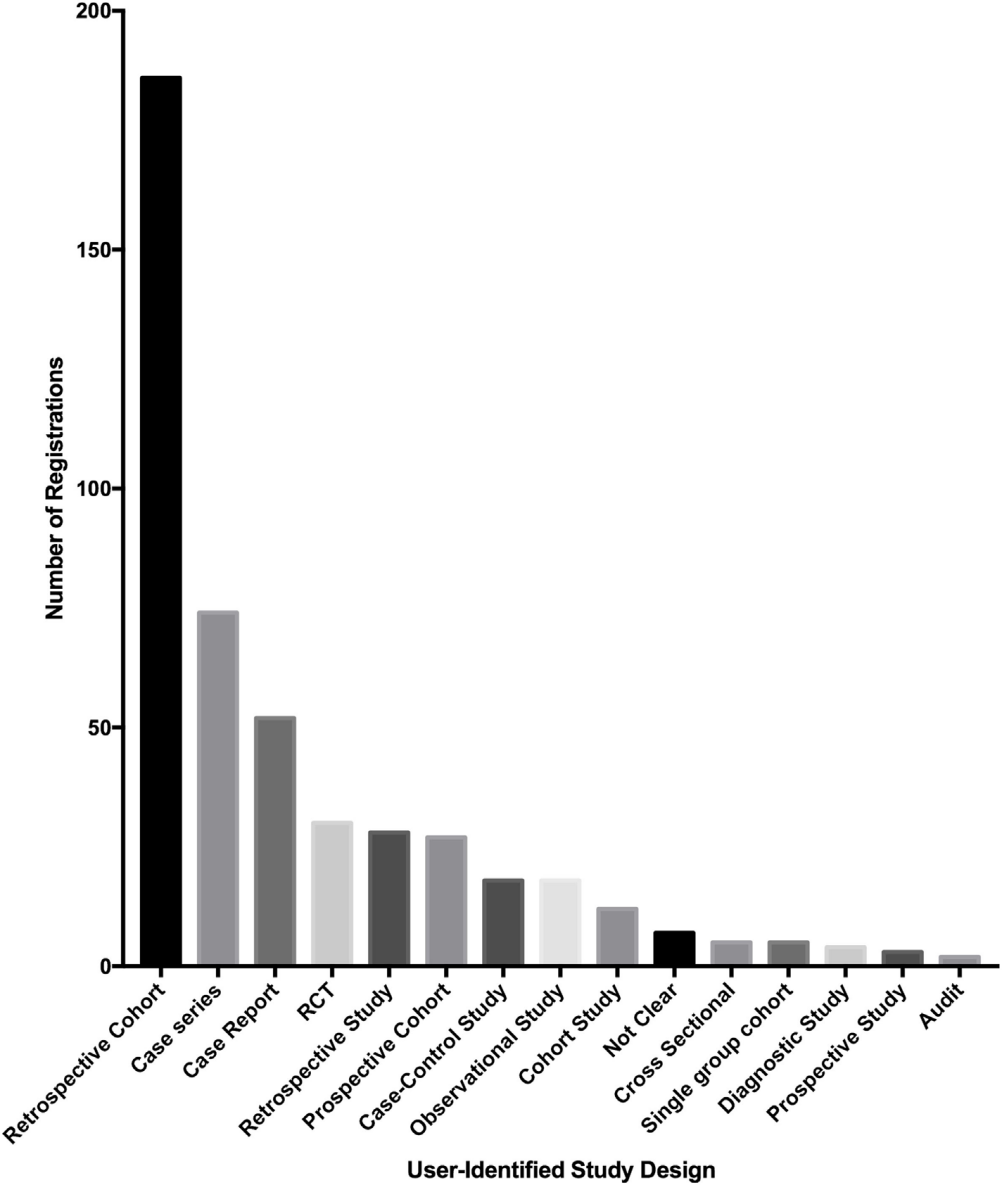
**Bar chart of the types of studies registered in the Research Registry^®^**.

Of the 500 studies registered to the Research Registry^®^, the commonest study type was retrospective cohort studies (186 of 500, 37.2%). Case series also made up a large proportion of registered studies (74 of 500, 14.8%). The third largest population was first-in-man case reports (52 of 500, 10.4%) (Figure 5).

### Data Curation

Retrospective weekly data curation resulted in 88 studies being deleted. Eighty studies were registered as the wrong study type (91% of 88 studies deleted) and there were eight duplicate studies that were deleted (9% of 90 studies deleted). These were studies that were inputted twice.

### Quality Criteria

**Figure 6:**
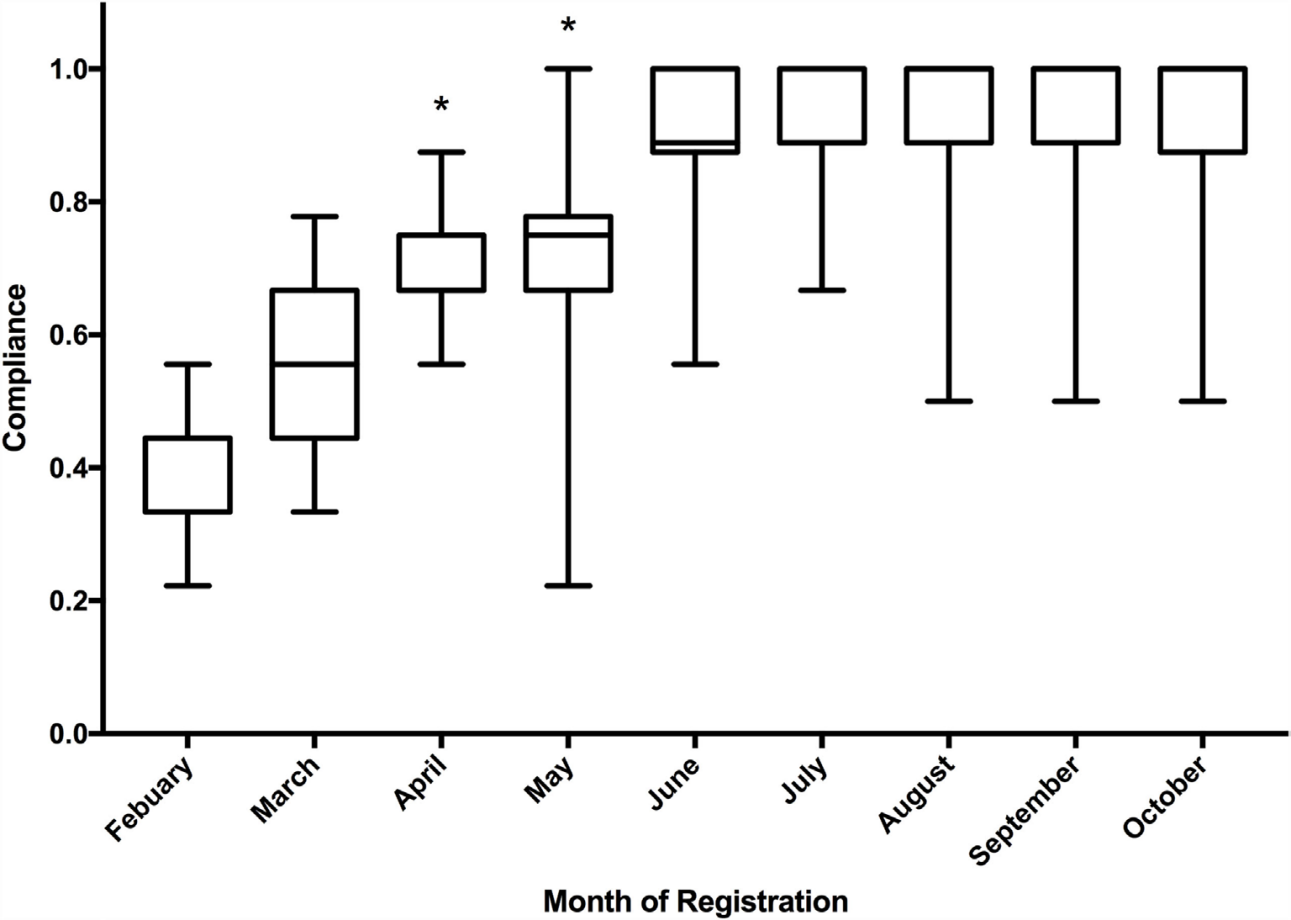
**Boxplot of median quality indicator score of registrations to the Research Registry^®^, including inter quartile range and minimum/maximum scores**. *Statistically significant according to Kruskal–Wallis test.

The quality criteria score increased significantly over the course of the first 500 registrations (Figure 6). The median quality score of the first 50 registrations was 44% (4 of 9) and gradually increased to 100% (9 of 9) for the last 50 registrations in the cohort of 500. When compared on a month-by-month basis, the median score improved month on month and the Kruskal–Wallis test demonstrated significant improvement in median quality scores over the study period (*p* < 0.001). The inter-rater agreement of quality scoring was 66.4% (a complete match of score was achieved in 332 of 500 scores).

## Discussion

Over the study period, the Research Registry^®^ has received registrations from over 57 countries, accounting for over 1.77 million patients, who were otherwise in unregistered studies. Registrations per month gradually increased over the study period, with the exception of August. Objective quality indicators have improved over the course of the first 500 registrations.

The highest number of registrations was received from Turkey, China, and the UK. This reflects in part submissions to two journals, IJS and Annals of Medicine and Surgery (AMS), where registration is mandatory promoting the Research Registry^®^ as a venue for registration. Concerns were initially raised as to whether clinicians would be willing to register their study as a necessary step during the submission process. This concern was unfounded and authors have engaged well with the process, with no drop in the number of manuscripts being received at IJS or AMS. The number of submissions deleted for being incorrectly registered (*n* = 80) was mostly case reports. The IDEAL framework to improve the quality of surgical research encourages the registration of first-in-man case reports (9). These should reflect the first time a particular technique is utilized clinically; however, some authors incorrectly register a normal case report with Research Registry^®^.

We have observed a gradual increase in registrations per month, from launch in February 2015, to the end of the study period in October 2015, with the exception of August. This is likely due to the reduced number of papers submitted for publication during August and, therefore, less authors directed to the registry. During the study period, over 1.77 million patients were included in registered studies and prior to the launch of the Research Registry^®^, many of these studies would not have had a venue of registration. The breadth of countries from which registrations have been received demonstrates the global impact of the Research Registry^®^ and the breadth of research registration that can be achieved.

It has been estimated that 10% of all observational studies are registered at present. When establishing the Research Registry^®^, observational studies were a key focus and this is evidenced in our study breakdown. Over 37% of the registrations during the study period were retrospective cohort studies – studies that previously would have limited options for registration, and if conducted retrospectively, no venue of registration, prior to the launch of the Research Registry^®^. Given other registries have found the registration of observational studies challenging, our example of utilizing a journal to boost compliance may be a way forward to improve registration of such studies. The registry has evolved from its surgical roots and recent registrations include an intervention to improve nutritional intake; the investigation of physical activity in women who have had treatment for breast cancer (10) and a pilot study of tremor disruption in Parkinson’s disease (11). Through an agile and iterative approach, new features have been added. These include unique features, such as the ability to upload multimedia to demonstrate interventions, for example, videos of surgical procedures. In September 2015, we added the function to deposit data and results, as well as the ability to update entries. We have also integrated key reporting criteria from STROBE, CONSORT, and PRISMA into our registration forms.

This analysis has a number of limitations, it is limited to a solitary database and we have had a number of challenges, which are similar to other established registries (12). Initially, we had difficulty with inappropriately filled fields in registrations and this reflects the poor quality score initially (Figure 6). This was improved with the combination of mandatory fields and careful data curation. Over time, the registration form has been improved both with formal reviews of the database, and with suggestions from data curators as they review registrations on a weekly basis. We have also experienced technical difficulties, such as the “search the database” function initially using a Google-based search, which was prevented by a national firewall in China. This was rectified by using a non-Google search facility and this aspect of the site has been functioning well in China since July 2015, and during the study period, China provided over 10% of registrations (13).

The Research Registry^®^ has established itself as a new registry with a clear focus on areas not well represented in existing registries, such as observational studies and those registering retrospectively. Going forward, our plan is to continue developing the platform in line with user and data curator feedback and usability studies. We also aim to establish ourselves as a novel registry, addressing the unmet demands of existing registries to include other study types. Ultimately, we will use this registry to improve compliance with the Declaration of Helsinki 2013.

## Author Contributions

All authors contributed substantially to the implementation of the study. Specifically, RA was involved in the conception and design of the study. RA, AF, CL, YA, HS, KK, DJ and MA were involved in data acquisition and analysis. RA, AF, CL, PM and DO were involved in data interpretation. Subsequently, RA wrote the first draft, and all authors were then involved in the critical revision and approval of the manuscript.

## Conflict of Interest Statement

The authors declare that the research was conducted in the absence of any commercial or financial relationships that could be construed as a potential conflict of interest.
